# Time-lapse imagery of Adélie penguins reveals differential winter strategies and breeding site occupation

**DOI:** 10.1371/journal.pone.0193532

**Published:** 2018-03-21

**Authors:** Caitlin Black, Colin Southwell, Louise Emmerson, Daniel Lunn, Tom Hart

**Affiliations:** 1 Department of Zoology, University of Oxford, Oxford, United Kingdom; 2 Department of Environment, Australian Antarctic Division, Channel Hwy, Kingston, Tasmania, Australia; 3 Department of Statistics, University of Oxford, Oxford, United Kingdom; Stockholms Universitet, SWEDEN

## Abstract

Polar seabirds adopt different over-wintering strategies to survive and build condition during the critical winter period. Penguin species either reside at the colony during the winter months or migrate long distances. Tracking studies and survey methods have revealed differences in winter migration routes among penguin species and colonies, dependent on both biotic and abiotic factors present. However, scan sampling methods are rarely used to reveal non-breeding behaviors during winter and little is known about presence at the colony site over this period. Here we show that Adélie penguins on the Yalour Islands in the Western Antarctic Peninsula (WAP) are present year-round at the colony and undergo a mid-winter peak in abundance during winter. We found a negative relationship between daylight hours and penguin abundance when either open water or compact ice conditions were present, suggesting that penguins return to the breeding colony when visibility is lowest for at-sea foraging and when either extreme low or high levels of sea ice exist offshore. In contrast, Adélie penguins breeding in East Antarctica were not observed at the colonies during winter, suggesting that Adélie penguins undergo differential winter strategies in the marginal ice zone on the WAP compared to those in East Antarctica. These results demonstrate that cameras can successfully monitor wildlife year-round in areas that are largely inaccessible during winter.

## Introduction

Extreme seasonality in the Polar Regions has forced many animals to develop adaptations to cope with a dynamic habitat, including a constrained breeding period and resilience during the non-breeding season. At the end of summer, most polar wildlife species enter a non-breeding, winter period with harsh environmental conditions in which individuals must survive while also building condition immediately prior to the next breeding season. Penguins (Spheniscidae) represent a large biomass of seabirds in the Southern Ocean during winter [1] but occupy different winter distributions [2–5] and undergo a range of strategies, depending on the species and colony [6]. King penguins (*Aptenodytes patagonicus*) breed during the summer months, but raise each chick for fourteen months over winter, resulting in an overlap in incubation and chick rearing each summer [7]. Similarly, other Southern Ocean species (eg. Adélie penguins, *Pygoscelis adeliae*; Chinstrap penguins, *Pygoscelis antarctica*; Gentoo penguins, *Pygoscelis papua*; Macaroni penguin, *Eudyptes chrysolophus*; Southern Rockhopper Penguin, *Eudyptes chrysocome*) hatch and raise their chicks to fledgings during summer, yet adopt various overwinter strategies to survive the non-breeding period and build condition prior to breeding the following summer [8].

These winter strategies include either long distance migrations (eg. Chinstrap, Adélie, and Southern Rockhopper penguins) or residence at the colony site (eg. Gentoo penguins, [9] and references therein) and depend on a complex relationship with sea ice [10–12], the ocean bathymetry and currents present [13–14], and prey availability [5,15]. For those that do migrate, ranges vary between species and colonies and species are often associated with either pack ice (eg. Adélie penguins) or open water (eg. Gentoo penguins) during winter, dependent on their frequency at the respective habitats and foraging strategies [10–11,16–17].

The *Pygoscelis* genus of penguins includes two species that migrate long distances (Chinstrap penguins [4,18] and Adélie penguins [19–21]) and one that stays predominantly at colony sites over winter (Gentoo penguins: see [22] and references therein), acting largely as a central place forager for the entire year. For those that do migrate, the duration and distance of migrations varies greatly by species and location, as colonies appear to winter in distinct regions to avoid an overlap in foraging areas [4,14,23]. An individual’s condition during the winter period may have major consequences for both sedentary and migratory species, affecting reproductive success during the following breeding period [24–27]. Subsequently, winter constraints, including a reduced photoperiod (aka. daylight hours) [12,28–30], sudden changes in sea ice types and locations [11], and dynamic prey movements [31], may influence whether an individual survives to return to breed in the summer [30,32] and an individual’s physical condition on return [33–34].

As an ice-obligate species during winter, recent evidence suggests that Adélie penguin populations have significantly declined along the Western Antarctic Peninsula (WAP) since 1980 [12,35–36], as their sea ice niche and therefore sea ice obligate prey has decreased due to less frequent cold years, while their populations have increased in East Antarctica where sea ice has remained relatively stable [37,38]. Consequently, monitoring of their populations is essential to track changes in the population dynamics and behaviors of this species year-round. As roughly 15% of adult Adélie penguins do not survive the winter months ([16] and references therein), it has been suggested that the non-breeding period serves as a critical time in the life history of the species, which likely dictates the stability of populations and recruitment rates within colonies [30,32,34–35].

Past studies examining Adélie overwintering behavior have primarily used tracking methods at individual colonies along East Antarctica, the Ross Sea [13,23] and along the Antarctic Peninsula [14,21,29,38]. From these studies, it appears that Adélie penguins in East Antarctica and the Ross Sea migrate large distances (~1400–17600 km; [13,19,23]), while studies along the WAP have found Adélies migrating more variable distances [14,21,39] with evidence that some individuals remain near the colony site over winter [14,40–42]. Unfortunately, tracking devices are often expensive and difficult to deploy long-term [43]; therefore, small sample sizes are often produced [19–20] without a complete winter season worth of information [19].

To further enhance knowledge of the overwintering behavior of Adélie penguins in the WAP and East Antarctica, we observed the presence of Adélie penguins at colony sites during the non-breeding period from camera images taken daily over winter in each of these regions. Cameras were deployed at the Yalour Islands along the WAP and at seven sites near Casey and Mawson stations in East Antarctica. Additionally, the cameras provided high-resolution data on sea ice extent at the Yalour Islands site, allowing us to accurately measure near-shore sea ice and its influence on Adélie penguin presence or absence at the colony site over winter. Using this information, we aimed to answer the following hypotheses:

1. Adélie penguins are present at the Yalour Islands breeding location and colony sites near Casey and Mawson stations throughout winter.
2. The number of adults present increases as sea ice extent diminishes and as daylight hours and daily air temperatures increase throughout the winter across the species’ range.

## Methods

### Study sites

**Fig 1:**
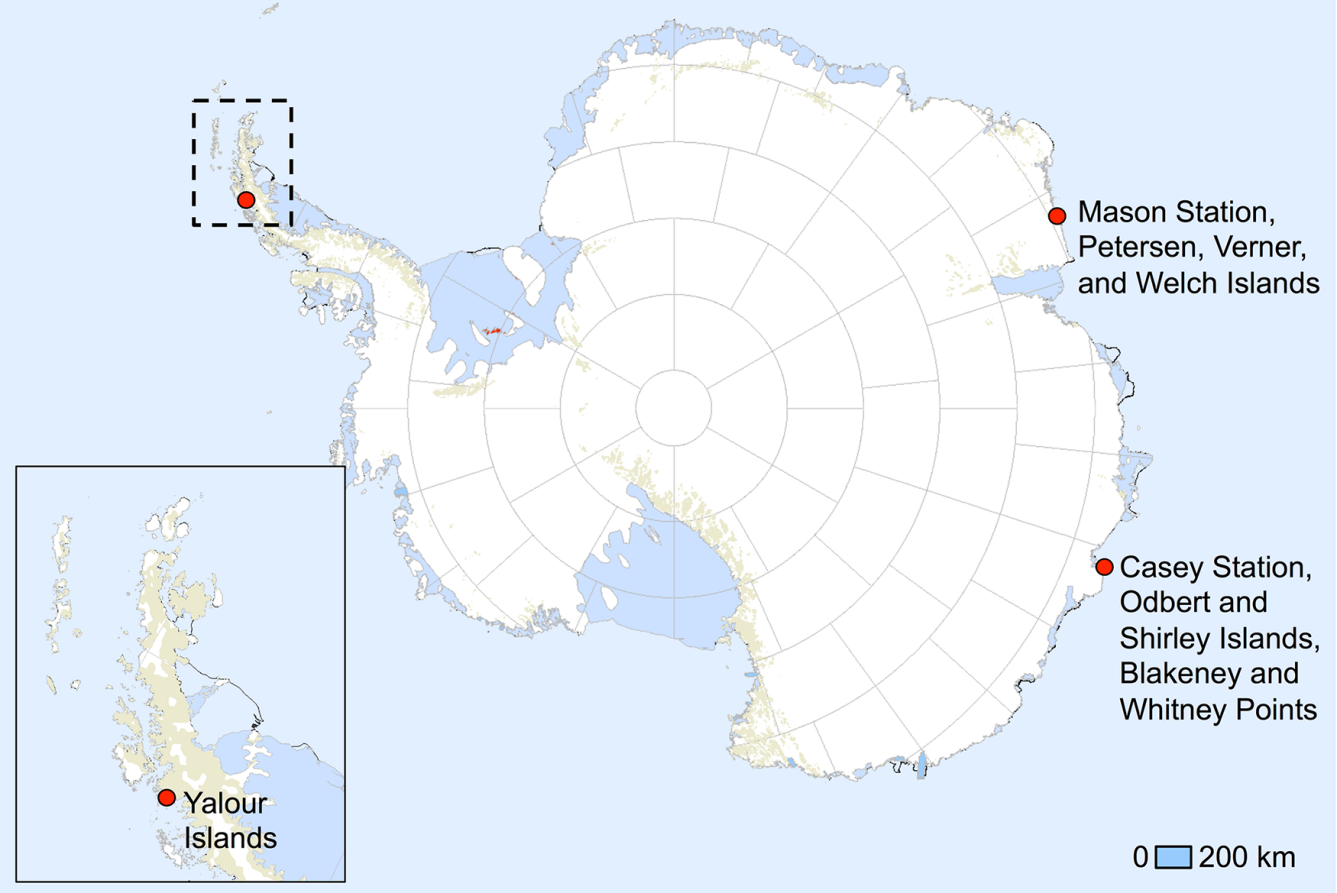
Map of our eight study sites, including 1) Yalour Islands on the Western Antarctic Peninsula, 2) Petersen Island, 3) Verner Island, 4) Welch Island, 5) Odbert Island, 6) Shirley Island, 7) Blakeney Point, and 8) Whitney Point in East Antarctica. Map adapted from USGS Antarctic Research Atlas [62].

Fieldwork was carried out under permits from the UK Foreign and Commonwealth Office and Australia’s Antarctic Treaty (Environmental Protection) Act 1980. Our study sites included Adélie penguin breeding sites in the WAP and in East Antarctica (Fig 1). In 2010, the Yalour Islands in the WAP (65°15’S, 64°17’W) were resident to 2555 Adélie penguin breeding pairs [44]. The group of three islands is positioned within the year-round marginal ice zone [35], which provides a variety of sea ice types throughout the year, dependent on local conditions. The study site is located on the northernmost main island and represents the species’ southern edge of range on the WAP [45].

The east Antarctic study sites comprised seven Adélie penguin breeding sites near Mawson and Casey stations that currently hold around 75,000 breeding pairs (Blakeney Point (66°25’ S, 110°53’ E), Odbert Island (66°24’ S, 110°58’ E), Shirley Island (66°28’ S, 110°48’ E), and Whitney Point (66°25’ S, 110°53’ E) near Casey Station; Petersen Island (67°57’ S, 62°88’ E), Verner Island (67°57’ S, 62°88’ E), and Welch Island (67°55’ S, 62°91’ E) near Mawson Station; Fig 1). These sites are on or close to (<2 km) the continental coastline where winter sea-ice can extend long distances northwards; winter pack-ice, in particular, can extend up to 1,000 km northwards in both areas [13]. Winter fast-ice, which can form a barrier to swimming, typically extends only short distances (2–10 km) northwards from the Casey sites, but can extend up to 80 km, northwards from the Mawson sites (NOAA National Ice Center 2015).

### Camera system

The Yalour Islands camera overlooked three sub-colonies of Adélie penguins. The camera angle was set to view the maximum number of individuals possible while still allocating a large portion of the camera view to the near-shore sea ice. The camera was installed using the techniques described by [46] with minor adjustments to the camera system. A Reconyx HC500 Hyperfire camera (Reconyx, Inc., Holmen, WI, USA) was installed and mounted to a scaffold pole and anchored using a rock basket. The camera was programmed in time-lapse mode to take eight photographs daily, beginning at 10:00 on March 1, 2012 and ending at 15:00 on January 6, 2014. The camera’s batteries (lithium AA) and SD card were changed twice, but the camera and its angle were not changed.

Similarly, each of the seven cameras deployed in East Antarctica were installed using the methods described in [46]. Each camera took one photo at solar midday each week (12:00 local time) from March 1 to September 27, 2013 and 2014 (31 total photos from each site per year). All cameras were deployed roughly 20–40 m from sub-colonies to include part of the breeding colony and four included the nearby coastline and ocean in the field of view. The SD cards were changed twice but the cameras’ positions were not changed.

### Data extraction

Counts were extracted from each image using software developed by [47]. In each image, a circle was centered over each visible individual to avoid counting individuals twice (S1 Fig). The number of circles in each image was then extracted, using the same software, to determine counts for each image date. For our study purposes, we defined winter as March 1- September 30 to provide a two-week buffer between the last departure date of fledgings from the previous breeding season and the first arrival date of adults the following spring. During the winter months, all birds were described as adults given the difficulty of confidently discerning adult and juvenile plumage due to the resolution (3.1 mpxl) of the images. The same individual (CB) annotated all images blind to date.

The Yalours camera on the WAP and four of the East Antarctica cameras (Odbert, Shirley, and Verner Islands and Whitney Point) overlooked the nearby marine environment enabling the extent and type of near-shore sea ice to be determined. Sea ice extent was determined from the images using the guidelines described in *An Observer’s Guide to Sea Ice* [48], originally intended for the U.S. Sea Ice Reporting Agencies’ observer reports forms. Sea ice in each image from all eight sites was ranked from 1–10 based off photographs provided in [48]. Rankings of 1–6 indicate increasing concentrations of open drift, rankings of 7–9 indicate increasing concentrations of pack ice, and a ranking of 10 indicates compact ice (aka. fast ice). We defined compact ice, separate from pack ice, when sea ice did not appear to move over time (between images).

All the cameras automatically produced metadata on date and time of day, and the Yalours camera also produced temperature data throughout the study period. Daylight hours were extracted using the *suncalc* function in the *RAtmosphere* package of R [49–50].

### Statistical analysis

We used generalized linear models (GLM) to model penguin abundance at occupied colony sites in relation to environmental conditions. To avoid bias due to low daylight hours during winter, and therefore a lower volume of visible counts around the winter solstice, we used only those images taken at 12:00 (local time). All analyses were conducted in R (v 3.0.3) language for statistical computing using the *stats* package [49], merging data from all years [S1 Table]. Due to overdispersion in both poisson and quasi-poisson generalized linear models, we used a negative binomial GLM (*glm*.*nb* function, *MASS* package; [51]) to determine interactions between each of the coefficients and non-breeding (winter) Adélie counts. By examining the model with and without a correlation structure and the auto-correlation in the observed residuals (*acf* function, *stats* package), we determined that temporal autocorrelation did not occur in our data set and a correlation structure was therefore omitted from the model. Model selection was determined by choosing the model with the lowest ratio of residual deviance to degrees of freedom. Ultimately, the GLM independent variables included an interaction between sea ice type and both photoperiod (aka. daylight hours) and temperature (°C), year, and Julian date (residual deviance: 238.92 on 618 degrees of freedom). During model selection, the log of the sea ice score (1–10) coefficient was omitted (ANOVA, p = 0.03). Lastly, to understand relationships with environmental variables, we examined contrasts by reordering the levels of factors (*relevel* function, *stats* package).

## Results

We observed considerable numbers of Adélies at the Yalour Islands colony site during the non-breeding (winter) period compared to those present during the breeding period. When birds were present during the non-breeding (winter) period, their abundance in the cameras’ field of view ranged from 1 to 13 in 2012, 1 to 29 in 2013, and 1 to 9 in 2014.

Analysis of images from the East Antarctic sites revealed that Adélies were last seen in photographs at the seven sites between February 28 and March 29 in 2013 and between 4 February and 27 February in 2014. There were no further sightings of penguins at any of the sites until the following October at the beginning of the next breeding season in both years. We therefore considered Adélies as absent from all of the East Antarctic colony sites over the entire non-breeding, winter period.

Because all East Antarctic sites were unoccupied over winter, our analysis of relationships between abundance and environmental conditions was restricted to the Yalours site. We found significant relationships between the abundance of Adélie penguins during the non-breeding (winter) period and each of the environmental variables tested in Table 1. Adélies were significantly more abundant during compact ice conditions (*P* = 0.015) than open water conditions (*P <* 0.001), although both sea ice types exhibited a significant relationship with non-breeding (winter) abundance (Table 1). The relationship between non-breeding (winter) abundance and photoperiod (aka. daylight hours) when compact sea ice was present (*P* = 0.038) was significantly different from photoperiod when open water was present (*P* < 0.001; Table 1). However, both open water and compact ice photoperiods influenced penguin abundance in a clear negative relationship (Table 1). In other words, penguins were more abundant at the colony site during the non-breeding (winter) period when fewer daylight hours and either high compact or low open water levels of sea ice were present. In addition, temperature during specific sea ice types also influenced Adélie abundance; the effect of temperature on penguin abundance when compact ice was present (*P* = 0.009) differed significantly from periods when open water (*P* = 0.17) or pack ice (*P* = 0.385) was present and exhibited a positive relationship with penguin abundance (Table 1). In other words, Adélie non-breeding (winter) abundance increased as temperature increased when compact ice was present.

**Table 1:** Results from a negative binomial GLM on Adélie penguin non-breeding (winter) abundance at the Yalour Islands from 2012–2014 (residual deviance: 238.92 on 618 degrees of freedom).

Coefficient		Estimate	SE	Z	P
Sea ice type	Open water	2.71	0.823	3.291	<0.001***
	Pack	3.26	1.884	1.730	0.084
	Compact	10.56	4.342	2.431	0.015*
Photoperiod: sea ice type	Photoperiod: open water	-0.20	0.578	-3.522	<0.001***
	Photoperiod: pack	-0.30	0.244	-1.250	0.211
	Photoperiod: compact	-1.04	0.503	-2.072	0.038*
Temperature (°C): sea ice type	Temperature: open water	-0.07	0.052	-1.372	0.170
	Temperature: pack	0.13	0.155	0.869	0.385
	Temperature: compact	0.82	0.315	2.615	0.009**
Year	2012	14.31	4.367	3.276	0.001***
	2013	-0.45	0.356	-1.276	0.202
	2014	-1.04	0.387	-2.692	0.007**
Julian date		-0.02	0.003	-4.326	<0.001***

*<0.05,

**<0.01,

***≤0.001

In relation to date, over all three years, Adélies were significantly more abundant earlier in the non-breeding (winter) season *(P <* 0.001). We also found significant differences between years; penguins were significantly more abundant in 2012 (*P* = 0.001) than in 2014 (*P* = 0.007), although there was no significant difference between 2013 and the other two years (*P* = 0.202; Table 1).

## Discussion

Here, we show that time-lapse cameras can successfully monitor colonial wildlife and near-shore sea ice conditions year-round, which will improve our understanding of winter behavior at colonies in remote regions. By using cameras, we can monitor areas that are logistically difficult to access and increase the replication of monitored sites [43]. Moreover, winter ecology in the Southern Ocean has largely used methods of non-constant effort, such as those in tracking studies (eg. [13–14,19–20]) or species assemblage surveys (eg. [10–11,29]) in which survey efforts are much lower and more constrained in winter when compared with the summer months. Camera monitoring demonstrates the strong potential of image analysis as a method to monitor colonial species and abiotic activities year-round at a high-resolution scale, overcoming many- but not all- limitations of other survey methods. However, the inabilities to 1) distinguish between adults and juveniles, 2) describe behaviors at night, 3) examine individuals outside of the camera’s range of view, and 4) know whether the individuals observed in winter at the Yalours also breed at the site serve as major limitations of this study’s camera method until more affordable technology develops.

Substantial differences exist between East Antarctica and the Western Antarctic Peninsula (WAP) in terms of environmental drivers, particularly related to winter sea ice extent. In East Antarctica, widespread consolidated compact ice exists in many regions throughout the winter months [52–55]. The presence of such ice is likely to make it difficult for an Adélie penguin to journey to the colony site during the non-breeding (winter) period without extensive, energy-consuming travel across the ice [16], as has also been observed during the summer months [56]. This may explain the winter absence of Adélie penguins from the Mawson colony sites, where winter fast-ice extends up to 1000 km from the coast [13]. Winter fast-ice extent is more limited at Casey, yet pack-ice can consolidate into large, dense floes over extensive areas in winter and form a physical barrier to travel in a similar fashion to compact ice. In contrast, the WAP sea ice extent varies substantially throughout the winter months, undergoing periods of open water, pack ice, and compact ice, with large temporal fluctuations [54]. Therefore, Adélies breeding on the WAP may either remain at their colony site during the winter period or choose to venture to other colony sites during this phase, without the limitations of seasonal compact sea ice extent.

Our results at the Yalours site support a strong association between winter sea ice and abundance; Adélies were present at the Yalour Islands during the non-breeding period when either compact sea ice or open water conditions were present (Table 1, Fig 1). Because Adélies are well-established pack ice inhabitants [30], they may not be able to exploit open-water areas as effectively due to adaptive restrictions (eg. diving behavior, temperature regulation; [10–11]), which may explain their increased presence on land during periods of open-water habitat offshore. Likewise, Adélies are likely restricted in their foraging abilities during short periods of compact ice extent when the sea ice forms a solid barrier from the land to sea. Therefore, the timing of winter presence at the colony site may reflect periods when Adélies are temporarily limited in their foraging abilities yet need a place to rest. Future studies should also consider whether sea ice conditions at a larger temporal scale, beyond the sea ice extent found directly offshore, influence Adélie winter abundance at a colony site.

**Fig 2:**
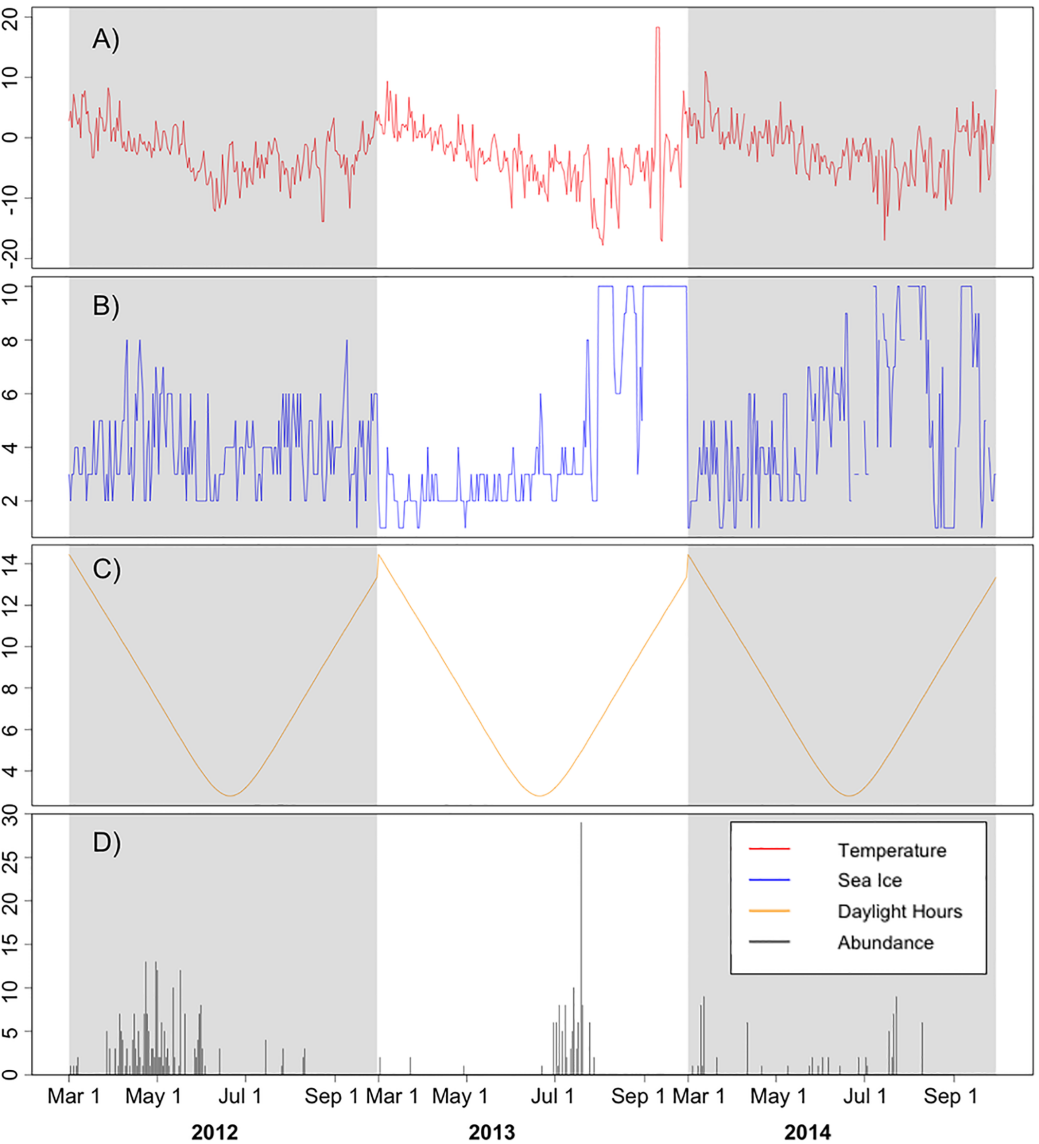
Non-breeding (winter) data at the Yalours site showing trends in (A) temperature, (B) sea ice rankings, (C) regional daylight hours, and (D) penguin abundance over time. Grey rectangles indicate the different study years: 2012, 2013, and 2014.

Our analysis on Adélies at the Yalours site also supported the idea that winter migrations may be linked with photoperiod (aka. daylight hours). As daylight hours decreased throughout the winter, Adélie non-breeding abundance at the colony increased when either open water or compact sea ice conditions were present offshore. Presence at the colony site during decreased photoperiods may indicate that penguins are resting on land (due to a the absence of visual cues necessary to navigate at sea), finding prey directly offshore, and avoiding predation during low light levels [56–60]. Adélie penguins at the Yalour Islands appear to peak in abundance once each non-breeding, winter period (Fig 2), which may reflect an individual’s need to forage closer to shore, when the amount of daylight hours is diminished and extreme sea ice extents (eg. low, open water or high, compact ice) are also present.

Our study highlights the lack of understanding of winter ecology, even though the winter months serve as a critical period of survival. With novel methods of constant effort, we are able to reveal a new mid-winter phase in the annual cycle of a colony at the edge of the species’ regional range, which would otherwise be limited by small sample sizes using survey efforts or tracking approaches. However, it is important to address that there is no way of knowing whether the birds in view during winter are the same as those in view over the summer months, yet it is assumed that many of the birds are present at both times of the year at the colony site based on observations of other Adélie populations in the WAP [14] and personal observations from the images studied here in which Adélies engage in nesting or nest building behavior during the non-breeding (winter) period. However, it is possible that many of those individuals seen overwintering at the colony site were first year fledglings. In addition, the observed individuals at the Yalour Islands, whether adult or juvenile, may have originated from other colonies and their presence may reflect anti-predatory behavior, or prospection of alternate available colony sites in addition to the hypotheses discussed. It is likely that our results describe the relationship between environmental variables and Adélie non-breeding abundance at the Yalours from colonies throughout the WAP as well as juveniles, rather than solely from adults breeding at the Yalours during the summer months. Future research should explore the age dynamics of overwintering birds as their presence or absence from the colony site over winter may affect their survival and therefore the overall recruitment within the colony.

It has been proposed that changes in climate over the coming years will increase competition for resources and habitat among the *Pygoscelis* species along the WAP [61]. This increased competition will likely be most apparent at the edge of their respective ranges and within the marginal ice zone. Future studies should directly address the degree to which the environmental conditions present during winter, particularly along a species’ edge of range, influence an individual’s reproductive success in the following season. Understanding winter behavior is essential to predicting population changes due to climate change in this rapidly warming region and understanding how other threats, such as nearby fishing of prey resources, will affect individual survival year-round.

## Supporting information

S1 Fig Camera-trap image from the Yalour Islands during the 2013 non-breeding period, demonstrating how Adélie penguin (*Pygoscelis adeliae*) counts were calculated. (TIFF)

S2 Fig Adélie penguin (*Pygoscelis adeliae*) abundance at median photoperiod (aka. daylight hours) and temperature (°C) for each of the three sea ice types observed: open water, pack, and compact. (TIFF)

S1 Table Raw data used during analyses of Adélie penguin (*Pygoscelis adeliae*) abundance at the Yalour Islands from 2012–2014 in.csv format. (CSV)
