# Supplementary figures and images for: Time-lapse imagery of Adélie penguins reveals differential winter strategies and breeding site occupation
Source: PLoS One. 2018 Mar 21;13(3):e0193532. doi: 10.1371/journal.pone.0193532 (PMC5862443; doi:10.1371/journal.pone.0193532)

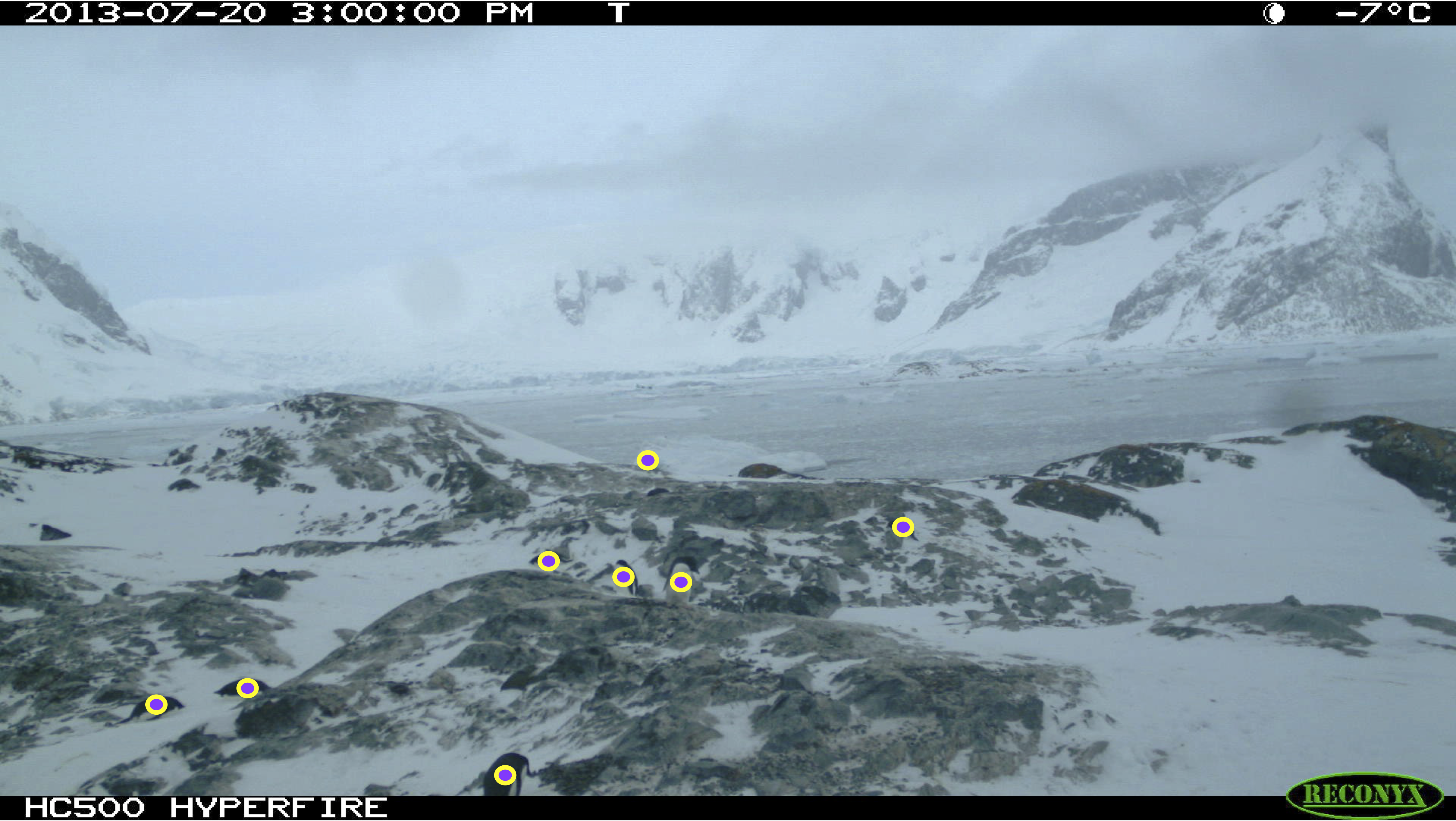

Supplement: S1 Fig — (TIFF) [file pone.0193532.s001.tiff]

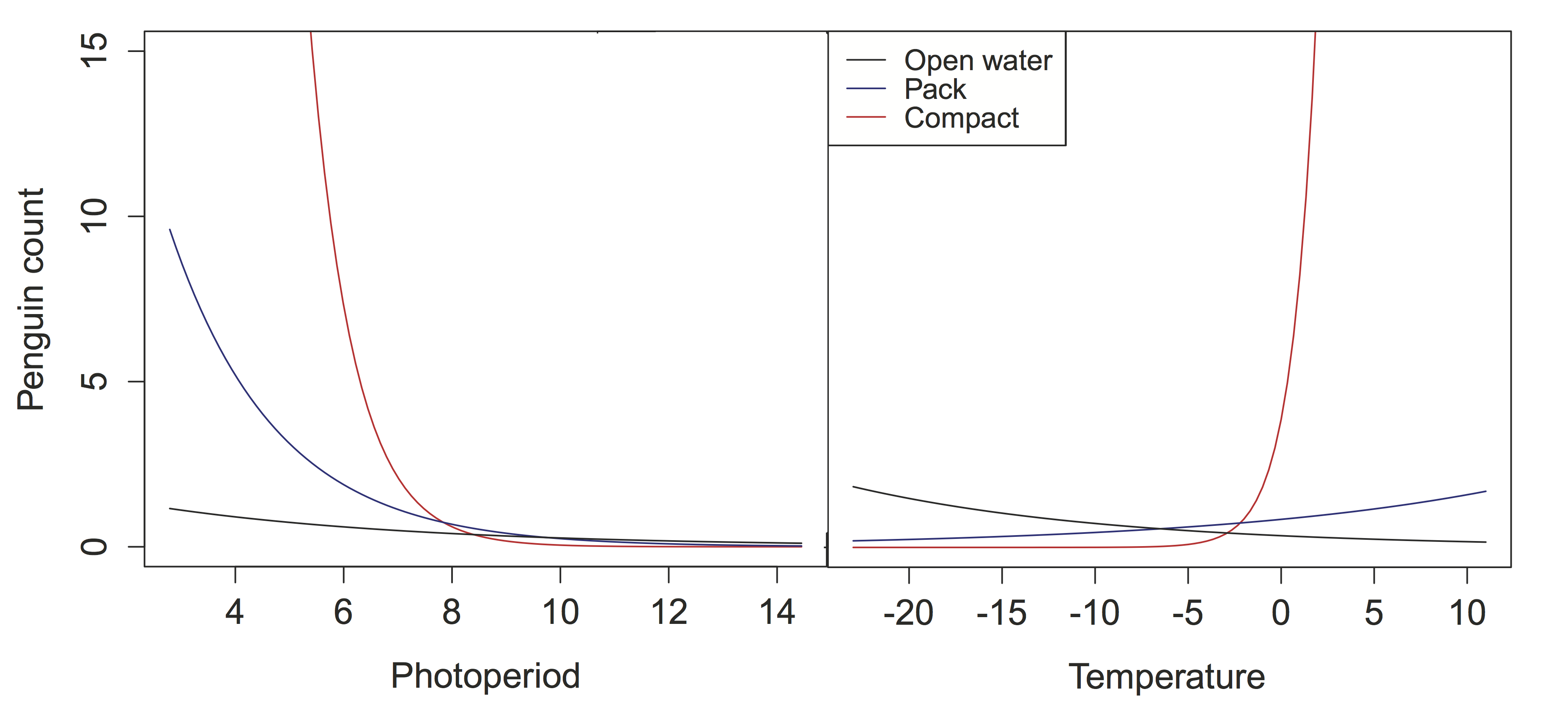

Supplement: S2 Fig — (TIFF) [file pone.0193532.s002.tiff]
